# Comparison of clinical and neuropathological diagnoses of neurodegenerative diseases in two centres from the Brains for Dementia Research (BDR) cohort

**DOI:** 10.1007/s00702-018-01967-w

**Published:** 2019-02-07

**Authors:** Sashika Selvackadunco, Katie Langford, Zohra Shah, Siobhan Hurley, Istvan Bodi, Andrew King, Dag Aarsland, Claire Troakes, Safa Al-Sarraj

**Affiliations:** 1 Department of Basic and Clinical Neuroscience, IoPPN, King’s College London, London, UK; 2 Old Age Psychiatry Department, IoPPN, King’s College London, London, UK; 3 Department of Clinical Neuropathology, Academic Neuroscience Centre, King’s College Hospital, King’s College Hospital NHS Foundation Trust, 1st Floor, Denmark Hill, London, SE5 9RS UK

**Keywords:** Brain banking, Neuropathology, Brain donation, Brains for Dementia Research, Clinical diagnosis, Clinicopathological correlation

## Abstract

Early detection and accurate diagnosis of neurodegenerative disorders may provide better epidemiological data, closer monitoring of disease progression and enable more specialised intervention. We analysed the clinical records and pathology of brain donations from 180 patients from two Brains for Dementia Research cohorts to determine the agreement between in-life clinical diagnosis and post-mortem pathological results. Clinical diagnosis was extracted from medical records and cases assigned into broad clinical groups; control, Alzheimer’s disease (AD), vascular dementia (CVD), dementia with Lewy bodies (DLB), frontotemporal dementia (FTD) and combined diseases. Pathology was assessed blindly, and cases categorised into; control, intermediate AD, severe AD, CVD, AD and CVD combined, DLB, AD and DLB combined and frontotemporal lobar degeneration (FTLD), according to the major contributing pathologies. In more than a third of cases clinical diagnosis was different from final neuropathological diagnosis. The majority of AD, DLB and control clinical groups matched the pathological diagnosis; however, thirty-five percent of clinical AD cases showed additional prominent CVD or DLB pathology which had not been diagnosed clinically and twenty-five percent of clinical control cases were found to have intermediate Tau pathology (modified Braak stage III–IV) or CVD. CVD and AD + CVD clinical groups showed an average of only thirty-two percent pathological correlation, the majority actually having no CVD, and fifty-three percent of pathologically identified FTLD cases had been incorrectly clinically diagnosed. Our results underlie the importance of neuropathological confirmation of clinical diagnosis. The relatively low accuracy of clinical diagnosis demonstrates the need for standardised and validated diagnostic assessment procedures.

## Introduction

Research shows that most people currently living with dementia have not received a formal diagnosis, with only 20–50% of dementia cases in high-income countries being formally recognised and documented in primary care settings (Alzheimer’s Disease International World Alzheimer Report 2011). This number is much greater in low- and middle-income countries.

Medical interventions and therapies are only available for those that have sought and received a diagnosis. Researchers are developing new drugs that may slow or stop the progression of the disease, particularly in the preclinical/early stage; however, trials of potentially preventative agents will only be possible once reliable methods of early diagnosis have been established. Increased reliability of diagnosis would also allow for the progression of the disease to be monitored more closely and provide an opportunity for more accurate determination of the specific dementia subtype. This would enable more specialised intervention and treatment at an earlier stage of the disease, before extensive neurochemical and neuropathological changes have occurred.

As 50% of people with mild cognitive impairment (MCI) later develop dementia, this would be a crucial group to monitor. The National Institute for Health and Care Excellence (NICE 2006) guidelines urge primary healthcare staff to refer people who show signs of MCI to memory assessment services and when these services identify people with MCI (including those without memory impairment) they are advised to offer followup assessments to monitor cognitive decline. To improve the accuracy of the diagnosis made, NICE guidelines suggest a diagnosis of dementia should only be made after a comprehensive assessment has taken place (NICE 2018). Healthcare professionals with expertise in differential diagnosis are encouraged to use international standardised criteria to obtain a more specific subtype of dementia diagnosis, they suggest encompassing various structural imaging techniques to aid in differentiation when appropriate (NICE 2006, 2018).

Dementia has many causes, the most common type being Alzheimer’s disease (AD), followed by vascular dementia (CVD), and dementia with Lewy bodies (DLB), then less common types such as frontotemporal dementia (FTD) and others. Though the guidelines provide much hope for better characterised diagnosis of dementia in the future, currently terms such as unspecified dementia or mixed AD and vascular dementia are commonly used, which clinically describe a wide range of symptoms associated with a decline in memory and cognitive function.

Whilst “dementia” can only be diagnosed clinically the subtypes, including Alzheimer’s disease, dementia with Lewy bodies and vascular dementia are clinicopathological entities requiring certain pathological characteristics for definitive diagnosis. The well-defined guidelines for such diagnoses include the BNE modified *Braak score* (Braak 2006), *NIA-ABC Scoring* (Alafuzoff 2009), *McKeith Lewy body disease Score* (McKeith 2005), and *VCING* (Skrobot 2016). However, without an accurate in-life clinical diagnosis and assessment data to validate the pathological findings we still have an incomplete picture of the disease.

Projects such as Brains for Dementia Research (BDR) have the unique quality of providing longitudinal clinical data alongside post-mortem brain tissue. BDR is a network of brain bank centres across England and Wales established in 2007. Through regular standardised clinical assessments, such as mini mental state examination (MMSE), participants are monitored to assess memory, lifestyle and behavior. BDR participants are assessed annually if they have a memory impairment or diagnosis of dementia, and every 2 years if they are a healthy control (Hayes 2016). The assessments do not contribute to any formal clinical diagnosis.

Once the participant has passed away the brain tissue is donated to BDR associated brain banks who are able to establish the post-mortem diagnosis; both clinical assessment details and medical notes contribute to the eventual neuropathological diagnosis. The pathological evaluations for both London and Cardiff BDR cohorts are carried out in the neuropathology department of King’s College London.

This study analysed the BDR cohort from the London and Cardiff centres to determine the agreeability and discrepancy between in life clinical diagnosis and eventual pathological results; and examine the reliability of GP/memory clinic diagnosis of dementia alongside the efficiency of BDR clinical assessments. The results may aid in future clinical assessments and interpretation of symptoms to enable more accurate and specific diagnoses of dementia types.

## Materials and methods

Consent for clinical assessment, autopsy, neuropathological assessment and research were obtained from all subjects and all studies were carried out under the ethical approval of the MRC London Neurodegenerative Diseases Brain Bank and the Brains for Dementia Research project. All BDR cases from the London and Cardiff cohorts received between 2009 and 2016 were reviewed. The clinical diagnosis of each participant and where the diagnosis was made (i.e., hospital, GP, memory clinic) was extracted from the medical records. The most recent BDR clinical assessment scores for each case were also examined.

### Clinical diagnosis

The vast majority of dementia cases were given a diagnosis by a hospital team (Table 1). As they were assessed in secondary care it can reasonably be presumed that NICE approved procedures were followed and that they received specialised assessments following referral from a GP.

**Table 1:** Origin of dementia diagnosis

Origin of dementia diagnosis	Frequency	Percent
No dementia diagnosis given-clinical control	56	n/a
GP	6	4.8
Hospital team	84	67.2
Memory service	20	16
Older adult MH	5	4
Unknown/unspecified	10	8
Total	180	100.0

The 180 cases were grouped into 8 broad clinical groups by the BDR study clinical co-ordinators (DA, SH) who were blind from any neuropathological findings: (1) control: no dementia diagnosis or evidence of cognitive change; (2) AD: Alzheimer’s disease diagnosis including late onset, early onset, unspecified AD; (3) CVD: vascular dementia or cerebrovascular disease (including small vessel disease); (4) AD + CVD: Alzheimer’s disease and vascular features described; (5) DLB: dementia with Lewy bodies; (6) AD and DLB: Alzheimer’s disease with additional features of dementia with Lewy bodies; (7) FTD: frontotemporal dementia; and (8) other or unspecified: e.g., paranoid schizophrenia, dementia without specified type.

Neuropathological findings were analysed and broadly grouped by neuropathologists (SAS, IB) who reviewed the microscopy reports of all cases independent of any clinical detail. Cases were categorised into specific groups depending on the type and degree of contributing pathologies present. Modified Braak score, NIA-ABC and CERAD score, VCING and McKeith Lewy body disease criteria were analysed to prioritise the pathological findings and correctly group the cases (Table 2).

**Table 2:** Outline of the pathological groups and definitions

Group	Description	Pathology score
CONTROL	No pathology, low or age-related pathology, and/or other minor pathology	BRAAK (BNE) Score 0–II CERAD Not AD-Low VCING No CVD-Low
IM AD: intermediate	Alzheimer’s disease type changes may or may not indicate cognitive decline; including those with secondary pathology e.g. amyloid angiopathy, TDP-43 pathology	BRAAK (BNE) Score III-IV CERAD Moderate-High VCING No CVD-Moderate
AD: severe	Severe Alzheimer disease changes indicating cognitive decline; including those with secondary pathology e.g. amyloid angiopathy, TDP-43 pathology	BRAAK (BNE) Score V-VI CERAD Moderate-High VCING No CVD-Moderate
CVD	Cerebrovascular disease, including small vessel disease	VCING Moderate-High BRAAK (BNE) Score 0-II CERAD Not AD-Low
AD + CVD	AD and significant CVD pathology	BRAAK (BNE) III-VI* CERAD Intermediate-High VCING Moderate-High
DLB	Dementia with Lewy Bodies; in absence of AD pathology	McKeith Limbic/ Neocortical stage DLB Braak stage: 5–6 Prob of DLB: Moderate-High BRAAK (BNE) 0-II
AD + DLB	AD and significant Lewy body pathology	McKeith Limbic/ Neocortical stage DLB Braak stage 5–6 Prob of DLB Moderate-High BRAAK (BNE) III-VI* CERAD Moderate-High
FTLD	Combined all frontotemporal lobar degeneration types, including **CBD, PSP and globular glial tauopathy (GGT)	Any FTLD subtypes
Other	Other diagnosis such as Argyrophillic grain disease (AGD), herpes simplex virus (HSV-1) and infarcts	

Neuropathological groups: *When AD is mentioned combined with CVD or DLB it includes both intermediate and severe AD pathology

**CBD and PSP were included in the FTLD pathological group- as although clinical presentation of these conditions could include Parkinsonian features there was no mention of these syndromes in the medical notes

Once all cases were grouped according to their clinical and pathological diagnoses, both data sets were combined. Each case was assessed to determine if the clinical diagnosis matched with the final neuropathological findings.

If the clinical and pathological group for a case did not match, the case was reviewed to establish whether this mismatch could be explained; the pathological reports were revisited in detail, as well as GP notes and further assessment records. This resulted in some originally non-matched cases subsequently being determined to match, based on clinical circumstances (e.g., pathology was a sudden event just before death) or accounting for factors such as age-related changes, which the pathology scores alone did not consider. Within the largest clinical groups (control and AD) the BDR assessment scores were statistically analysed using an independent sample *T* test to identify any trends or significant differences in values between matching and non-matching cases.

## Results

### Correlation between clinical and neuropathological diagnosis

**Fig. 1:**
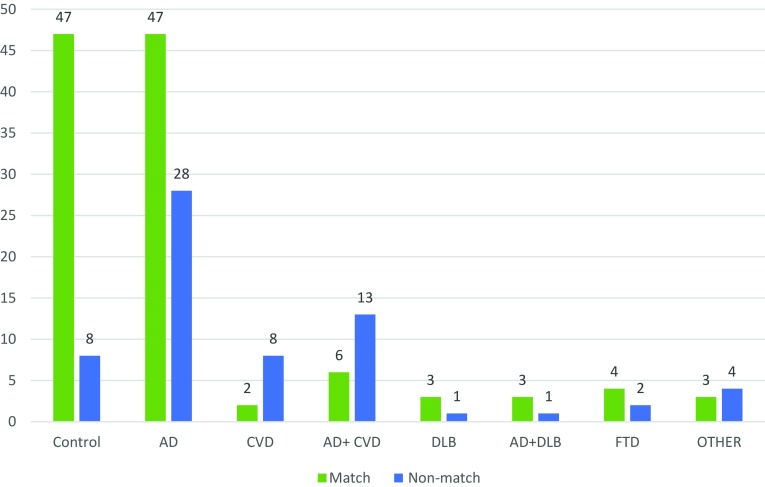
The number of matching and non-matching cases in each clinical group

Clinical and neuropathological diagnosis was determined to match in only 115 of the 180 cases examined (Fig. 1). Only 37 out of the 75 clinical AD cases were a direct match pathologically, with most of these cases being classified as severe AD (modified Braak stage V–VI), a further 26 of these clinical AD cases had coexisting DLB or CVD pathology that had not been detected clinically (Table 3). Seven of the clinical AD cases were determined pathologically to be FTLD. The CVD and AD + CVD clinical groups had a wider range of different pathological diagnoses, though many of these had either severe AD pathology or mixed pathology of either AD + CVD or AD + DLB.

**Table 3:**
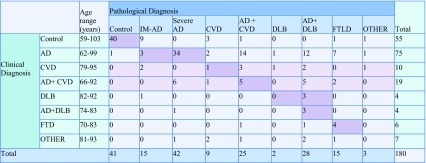
Cross tabulation of clinical diagnosis and final neuropathological diagnosis

The majority (3/4) of the cases with a clinical DLB diagnosis were matched with AD + DLB pathology, with the remaining case being diagnosed as AD. In the clinical FTD group, four out of six of the cases were pathologically matched with FTLD, the other two cases were diagnosed as AD + DLB and AD + CVD. Within the clinical control group, 40 out of the 55 participants were determined to be controls pathologically (i.e., no or very minor pathology present); however, the remaining 15 cases were found to have significant pathology despite no report of cognitive decline (Table 3).

The number of matching and non-matching cases in each clinical group was recalculated after further review of the cases to account for any explanation of the mismatch (see “Materials and methods”) and the pathology groups were reorganised by splitting cases from the intermediate AD group into either control and AD depending on the whether the pathology present was deemed to be normal age-related changes, or indication of Alzheimer’s disease progression. Percentage matches within each of the clinical diagnosis groups were then calculated (Table 4), although we excluded the ‘other’ group from further analysis due to the diversity of diagnosis within the group and the inability to match these appropriately.

**Table 4:** Percentage of match and non-matched cases within each clinical diagnosis groups

Clinical diagnosis	Matched (%)	Non-matched (%)
Control	85.5	14.5
AD	62.7	37.3
CVD	20.0	80.0
AD + CVD	31.6	68.4
DLB	75.0	25.0
AD + DLB	75.0	25.0
FTD	66.7	33.3
Total	64.4	35.6

Overall 64% of all cases were deemed to match clinically and pathologically. The control, AD, DLB, AD + DLB and FTD clinical groups had a majority of cases matching; however, the CVD and AD + CVD group were poorly matched with only 20–32% of the cases matching.

### Clinical assessment results

Table 5 shows 123 cases, where MMSE assessments had been carried out within 4 years prior to death. Based on NICE guidelines BDR considers a score of nine or under as showing severe impairment of cognitive function, 10–20 as moderate impairment of cognitive function, 21–24 as mild impairment of cognitive function and a score of 25 to the maximum 30 would be considered normal (NICE 2001).

**Table 5:** 123 of the 180 cases with recent MMSE scores

MRC ID	Age at death	Gender	MMSE score	MMSE out of	Percentage score (%)	Coded MMSE	Impairment of cognitive functioning	Clinical Diagnosis Group
BBN_25640	95	F	24	26	92.31	0	Normal	CONTROL
BBN002.26316	96	F	25	30	83.33	0	Normal	CONTROL
BBN002.29633	96	F	26	30	86.67	0	Normal	CONTROL
BBN_24297	83	F	27	30	90.00	0	Normal	CONTROL
BBN002.30036	89	F	28	30	93.33	0	Normal	CONTROL
BBN_21005	77	F	28	30	93.33	0	Normal	CONTROL
BBN002.29730	91	F	28	30	93.33	0	Normal	CONTROL
BBN_10199	93	F	28	30	93.33	0	Normal	CONTROL
BBN_20012	87	F	29	30	96.67	0	Normal	CONTROL
BBN_20194	102	F	29	30	96.67	0	Normal	CONTROL
BBN_16210	89	F	29	30	96.67	0	Normal	CONTROL
BBN_9948	86	F	29	30	96.67	0	Normal	CONTROL
BBN_11073	90	F	29	30	96.67	0	Normal	CONTROL
BBN_25743	73	F	29	30	96.67	0	Normal	CONTROL
BBN_4578	90	F	30	30	100.00	0	Normal	CONTROL
BBN_25987	98	F	30	30	100.00	0	Normal	CONTROL
BBN_24243	69	F	30	30	100.00	0	Normal	CONTROL
BBN_16233	88	F	30	30	100.00	0	Normal	CONTROL
BBN_16217	85	F	30	30	100.00	0	Normal	CONTROL
BBN002.29498	75	F	30	30	100.00	0	Normal	CONTROL
BBN_24215	91	F	30	30	100.00	0	Normal	CONTROL
BBN002.28350	75	M	25	30	83.33	0	Normal	CONTROL
BBN_18399	77	M	26	30	86.67	0	Normal	CONTROL
BBN_23398	84	M	26	30	86.67	0	Normal	CONTROL
BBN002.26065	74	M	27	30	90.00	0	Normal	CONTROL
BBN_10593	86	M	28	30	93.33	0	Normal	CONTROL
BBN_25741	87	M	28	30	93.33	0	Normal	CONTROL
BBN002.26311	81	M	28	30	93.33	0	Normal	CONTROL
BBN002.28858	80	M	28	30	93.33	0	Normal	CONTROL
BBN_19209	82	M	28	30	93.33	0	Normal	CONTROL
BBN_4582	95	M	29	30	96.67	0	Normal	CONTROL
BBN002.26151	83	M	29	30	96.67	0	Normal	CONTROL
BBN002.29089	81	M	29	30	96.67	0	Normal	CONTROL
BBN_13802	74	M	29	30	96.67	0	Normal	CONTROL
BBN002.28121	80	M	29	30	96.67	0	Normal	CONTROL
BBN002.29416	88	M	29	30	96.67	0	Normal	CONTROL
BBN_24576	92	M	29	30	96.67	0	Normal	CONTROL
BBN_10204	87	M	29	30	96.67	0	Normal	CONTROL
BBN_16213	74	M	30	30	100.00	0	Normal	CONTROL
BBN_10610	78	M	30	30	100.00	0	Normal	CONTROL
BBN002.28769	91	M	25	29	86.21	0	Normal	AD
BBN_16230	80	F	26	30	86.67	0	Normal	AD
BBN_15199	89	F	28	30	93.33	0	Normal	AD
BBN002.29617	83	M	26	30	86.67	0	Normal	AD
BBN_16231	83	M	25	30	83.33	0	Normal	FTD
BBN002.29836	84	F	28	30	93.33	0	Normal	CVD
BBN_24680	82	M	26	30	86.67	0	Normal	CVD
BBN_20618	86	F	30	30	100.00	0	Normal	AD + CVD
BBN002.29535	83	M	25	30	83.33	0	Normal	AD + DLB
BBN_10599	85	F	28	30	93.33	0	Normal	Other/unspecified
BBN_19696	93	M	28	30	93.33	0	Normal	Other/unspecified
BBN_16199	85	M	15	21	71.43	1	Mild	CONTROL
BBN_10611	92	F	17	24	70.83	1	Mild	CONTROL
BBN002.29843	103	F	20	27	74.07	1	Mild	CONTROL
BBN_18804	88	M	23	30	76.67	1	Mild	AD
BBN002.26278	74	M	24	30	80.00	1	Mild	AD
BBN002.28945	81	F	21	30	70.00	1	Mild	CVD
BBN002.28123	87	M	21	30	70.00	1	Mild	DLB
BBN_10191	84	M	22	30	73.33	1	Mild	Other/unspecified
BBN_24246	91	F	13	30	43.33	2	Moderate	CONTROL
BBN002.29910	68	F	11	30	36.67	2	Moderate	AD
BBN_9919	91	F	13	30	43.33	2	Moderate	AD
BBN_25890	76	F	14	30	46.67	2	Moderate	AD
BBN002.29132	90	F	14	30	46.67	2	Moderate	AD
BBN_9944	84	F	15	30	50.00	2	Moderate	AD
BBN_10598	90	F	16	30	53.33	2	Moderate	AD
BBN_15195	84	F	18	30	60.00	2	Moderate	AD
BBN002.28925	87	M	10	30	33.33	2	Moderate	AD
BBN_21800	82	M	11	30	36.67	2	Moderate	AD
BBN002.28543	96	M	12	30	40.00	2	Moderate	AD
BBN_9900	88	M	17	30	56.67	2	Moderate	AD
BBN_18800	90	M	18	30	60.00	2	Moderate	AD
BBN_21792	87	M	19	30	63.33	2	Moderate	AD
BBN002.29842	82	M	19	30	63.33	2	Moderate	AD
BBN_9938	72	M	11	30	36.67	2	Moderate	FTD
BBN_16229	79	M	18	30	60.00	2	Moderate	CVD
BBN002.28665	86	M	18	30	60.00	2	Moderate	CVD
BBN_9918	88	F	16	30	53.33	2	Moderate	AD + CVD
BBN_24369	91	F	18	30	60.00	2	Moderate	AD + CVD
BBN_20991	83	M	11	30	36.67	2	Moderate	DLB
BBN_9940	67	M	20	30	66.67	2	Moderate	other/unspecified
BBN_9984	79	M	0	16	0.00	3	Severe	AD
BBN_24356	80	F	5	20	25.00	3	Severe	AD
BBN002.26315	93	F	0	28	0.00	3	Severe	AD
BBN_16234	88	F	0	30	0.00	3	Severe	AD
BBN_15196	94	F	0	30	0.00	3	Severe	AD
BBN_9981	95	F	0	30	0.00	3	Severe	AD
BBN002.29877	99	F	0	30	0.00	3	Severe	AD
BBN_24938	87	F	0	30	0.00	3	Severe	AD
BBN_18400	94	F	0	30	0.00	3	Severe	AD
BBN_22595	94	F	0	30	0.00	3	Severe	AD
BBN_16226	91	F	0	30	0.00	3	Severe	AD
BBN_19999	77	F	0	30	0.00	3	Severe	AD
BBN_15301	87	F	2	30	6.67	3	Severe	AD
BBN_14413	98	F	7	30	23.33	3	Severe	AD
BBN_20621	89	M	0	30	0.00	3	Severe	AD
BBN_24944	81	M	0	30	0.00	3	Severe	AD
BBN_25642	95	M	0	30	0.00	3	Severe	AD
BBN_23391	84	M	0	30	0.00	3	Severe	AD
BBN_20018	83	M	0	30	0.00	3	Severe	AD
BBN_16228	96	M	0	30	0.00	3	Severe	AD
BBN_20003	62	M	0	30	0.00	3	Severe	AD
BBN_9929	70	M	0	30	0.00	3	Severe	AD
BBN_24939	86	M	0	30	0.00	3	Severe	AD
BBN_16207	88	M	0	30	0.00	3	Severe	AD
BBN_15304	80	M	0	30	0.00	3	Severe	AD
BBN002.29900	86	M	0	30	0.00	3	Severe	AD
BBN002.29053	71	M	2	30	6.67	3	Severe	AD
BBN_9972	92	M	7	30	23.33	3	Severe	AD
BBN002.29071	94	M	7	30	23.33	3	Severe	AD
BBN_24550	85	M	8	30	26.67	3	Severe	AD
BBN_13796	81	M	8	30	26.67	3	Severe	AD
BBN_24199	84	M	9	30	30.00	3	Severe	AD
BBN002.26717	70	F	0	30	0.00	3	Severe	FTD
BBN_24551	74	M	0	30	0.00	3	Severe	FTD
BBN002.29499	74	M	0	30	0.00	3	Severe	FTD
BBN_10206	87	F	4	30	13.33	3	Severe	CVD
BBN002.28117	95	F	8	30	26.67	3	Severe	CVD
BBN_19996	76	F	3	25	12.00	3	Severe	AD + CVD
BBN_24202	86	F	0	30	0.00	3	Severe	AD + CVD
BBN002.29634	73	M	0	30	0.00	3	Severe	AD + CVD
BBN002.28700	74	M	2	16	12.50	3	Severe	AD + DLB
BBN002.29411	80	M	0	30	0.00	3	Severe	AD + DLB

Table 6 shows the spread of the most recent MMSE scores between diagnosis groups. As not all the assessments taken had been scored up to the full 30 points (e.g., patient was unable to complete all the tasks), percentage scores were used to group the cases between cognitive states. Normal (> 83%), mild impairment of cognitive function (70–82%), moderate impairment of cognitive function (33–69%), and severe impairment of cognitive function (< 32%). The control group, as expected, had the highest mean score for MMSE. The mean scores for FTD and AD cases were significantly lower than all other conditions which suggests the MMSE scoring system may be more able to identity FTD and AD, and that diagnosis of these conditions may be aided by the more obvious presentation of cognitive decline via MMSE assessments.

**Table 6:** Spread of the most recent MMSE scores between diagnosis groups

Clinical diagnosis group	Mean MMSE score	Mean percentage score	Number of cases	Normal cognitive state 25–30	Mild impairment of cognitive functioning 21–24	Moderate impairment of cognitive functioning 10–20	Severe impairment of cognitive functioning < 9
Control	27/30	90.0	44	40	3	1	0
AD	8/29	27.6	52	4	2	14	32
FTD	7/30	23.3	5	1	0	1	3
CVD	18/30	60.0	7	2	1	2	2
AD + CVD	11/29	37.9	6	1	0	2	3
DLB	16/30	53.3	2	0	1	1	0
AD + DLB	9/25	36.0	3	1	0	0	2
Other	25/30	83.3	4	2	1	1	0
Total			123	51	8	22	42

An independent sample *T* test was carried out on the clinical ‘control’ group and clinical ‘AD’ group to determine if there was any significant difference between the matched and non-matched cases, and to identify any trends in the BDR Clinical assessments (MMSE, NPI, GDS, CDR scores) across the groups. No significant difference was found in any of the clinical assessment scores between the matched and non-matched cases in either clinical group.

## Discussion

Analysis of the diagnosis in the London and Cardiff BDR cohort demonstrates that there are a large proportion (36%) of cases that show a different pathological diagnosis to the one given clinically.

25% (14 out of 55) of the clinical control group exhibited significant pathology. If these cases were in the preclinical phase of AD, as describe by Jack et al. (2011), presentation of clinical symptoms may have been absent. The cases could alternatively have been in the pre-dementia or MCI phase of AD, as described by Sperling et al. (2011), and any symptoms presented too subtle to indicate a dementia diagnosis. This is supported by the BDR clinical assessment data, which did not show any significant difference in assessment scores between matched and non-matched controls.

Age is vital to consider when diagnosing a patient with AD. Price et al. (2009) examined a group of cognitively normal people and found that neuropathological processes related to AD in persons without dementia appear to be associated with subtle cognitive dysfunction representing a preclinical stage of the illness (2009). Interestingly, by the age of 80–85 years, as many as 40% of non-demented cases demonstrated AD pathology. With strict criteria applied approximately 20% of cases had AD pathology. In our study 16% (9 out of 55) of clinical controls had moderate AD pathology (i.e., modified Braak stages III–IV); up to 20% when the 3 CVD and 1 FTLD cases are included, which had accompanying Braak stage III–IV pathology.

During the re-analysis, the neuropathologists determined that in five out of the nine cases originally scored as modified Braak stage III–IV there had been artificial elevation of the apparent modified Braak stage by actual age-related changes (Crary 2014) which would not have presented as clinical dementia; these cases were, therefore, no longer considered to be mismatched. The other four cases were not thought to be normal aging changes; therefore, we considered these mismatched cases. Someone with moderate AD pathology may or may not present with clinical symptoms of dementia thus making it difficult for a GP or care giver to recognise. Subtle cognitive changes may be missed by the standardised clinical assessments.

Findings from the ‘Nun Study of Aging and Alzheimer’s Disease’ also suggests that pathology can be present in the absence of clinical symptom presentation (Snowdon 1997). This continuing longitudinal study produced clinical and neuropathological data on 498 Catholic sisters, who all lived in relative homogeneity in terms of environment and lifestyle. 12% of participants who were not demented were found to have Braak stages V–VI pathology (SantaCruz 2011). An especially interesting case of an 85 years with well-preserved cognitive and physical function with a genetic predisposition to Alzheimer’s disease and an abundance of Alzheimer’s disease lesions was found (Snowdon 1997). This study provides useful information on how the degree of pathology present in the brain and the level of resistance to the clinical expression of the neuropathology can occur in some cases.

The nun study also found 29% of participants with dementia exhibited Braak stage II pathology or less (SantaCruz 2011). In the BDR cohort one case was diagnosed clinically as AD and presented symptoms of AD, such as significant problems in memory, irritable behavior and personality changes. Additionally, the brain scans indicated frontal lobe shrinkage. However, the only neuropathology observed in this case was minimal AD pathology Braak stage II and sparse AGD (argyrophilic grain disease) pathology. The microscopy report noted that it was difficult to explain the cognitive decline with the pathology presented. This is rare, however, not unheard of.

Nolan et al. (2015) demonstrated the importance of thorough neuropathological assessment on control tissue. This study re-investigated tissue donated prior to 2007, when new histological staining protocols for examining control brain were introduced. Almost all cases that were originally described as neuropathologically normal displayed some level of pathology after re-analysis. Four cases displayed ‘major’ pathological features which are usually associated with significant clinical features of dementia. This finding emphasises the importance of accurate neuropathological analysis of control tissue and highlights the inherent difficulty of classifying tissue as ‘control’.

In the clinical AD group 37% did not directly match pathologically. The majority of these cases had Alzheimer’s disease pathology but with additional prominent DLB or CVD pathology (fulfilling a combined diagnosis). This suggests that on the clinical side, diagnostic teams may not be detecting additional ‘DLB’ and ‘CVD’ clinical symptoms via the various assessment measures. The symptoms occurring in CVD, such as unusual movements, falls, problems with planning and organising, decision making or problem solving were not picked up by the current assessment methods (Jellinger 2008). Symptoms such as memory loss or confusion, which are more commonly associated with AD, are better addressed in the assessments. Both these symptoms are also commonly seen in CVD, which does make distinguishing CVD from AD difficult for assessors and GPs. With AD being the most common type of dementia, it seems GPs and assessors are more likely to diagnose people with AD. Brenowitz et al. (2017a, b) investigated whether associations between clinical progression and AD neuropathology were modified by co-occurring Lewy body disease (LBD). They found overall AD + LBD was associated with faster rate of progression than AD only, it was most evident in cases with intermediate AD pathology. Patients with DLB also have additional clinical symptoms such as hallucinations and parkinsonism and a worse prognosis than pure AD (Mueller 2017) and, therefore, often require specific management.

Our results are reflective of data collected from nine brain bank centres within the BrainNet Europe consortium (3303 cases) which showed 53.3% of dementia cases had a mixed pathological diagnosis (Kovacs 2008). This correlates with a number of other studies emphasising the contribution of mixed brain pathologies to dementia cases (Kovacs 2013; Mendez 1992; Robinson 2018).

Monitoring cognitive decline from an early point and applying diagnostic procedures to increase detection of dementias other than AD may highlight those who may benefit from treatments targeting multiple aetiologies.

The ‘CVD’ clinical group was poorly matched, only one case was a complete match clinically and pathologically. Three out of ten in this group had AD + CVD mixed pathology. The ‘AD + CVD’ clinical group had most of the cases mismatching, only 5 of the 19 in this group were a complete match. 6 of the 19 were pathologically diagnosed with AD in the absence of additional ‘CVD’ pathology. We utilised VCING scores to determine the level of vascular involvement in contributing to the dementia, we also looked at past medical records to see if any vascular changes were clinically observed and recorded. Seven cases in the group had no vascular neuropathology at all. This would suggest clinicians may be over-diagnosing patients with mixed AD and vascular dementia; when there is no clinical or pathological evidence of a vascular component. Brunnstrom et al. (2009) found similarly poorly matched CVD (59%) and mixed AD + CVD (25%) cases in their clinicopathological concordance study in Sweden. This is important, since it may lead to these patients not being treated with anti-dementia drugs such as ACE and memantine.

We can conclude that the clinical assessment procedures are not designed to pick up on an additional ‘CVD’ or ‘DLB’ component of AD type dementia (whereas CVD on its own is over-diagnosed). Statistical analysis showed there was no significant difference in the clinical assessment scores between the matched and mismatched cases. This could be due to AD symptoms masking the additional presentation of ‘CVD’ and ‘DLB’ symptoms, therefore, not being picked up efficiently during the diagnosis and assessment. When dementia patients are in the last stages of the dementia, quite often the symptoms presented are severe cognitive decline and memory loss, and inability to take care of oneself. If a patient is only diagnosed during this late stage, assessors may have missed other possible clinical presentations such as hallucinations, apathy and psychiatric syndromes, which may have eluded to the subtype of dementia.

In the clinical FTD group, four out of six cases were pathologically diagnosed as having FTLD. Correct identification of clinical ‘red flags’ for FTD may be responsible for the relatively high number of matched cases in the group. Behavioral or personality changes in the absence of prior psychiatric history often appears first; language decline characterised by impaired speech production or impaired word comprehension and semantic memory is also a key feature of the condition (Warren 2013). Memory, navigational skills and other aspects of general intellect are often well maintained in FTD patients initially. More widespread cognitive deficits like AD phenotypes emerging later in disease course. Hence the importance of early detection of the condition.

Though FTD is substantially less common than AD, the prevalence of FTD in older age groups has been almost certainly been underestimated (Warren 2013). Echavarri et al. (2012) investigated the agreement between clinical and neuropathological diagnoses of dementia cases donated to the Brain Bank of Navarre, Spain and found the agreement in FTLD (28%) to be much lower than the other dementia subtypes examined. In the BDR cohort we pathologically identified 15 FTLD cases; 7 of these cases were clinically diagnosed with AD and 2 cases were clinically diagnosed with AD + CVD. This suggests clinically FTD is being under diagnosed and misdiagnosed as AD or AD + CVD. This could be due to patients not being seen until later in the disease progression when symptoms of memory and cognitive decline have emerged and mask the more unique FTD symptoms. New consensus diagnostic criteria for FTD and other progressive aphasias have recently been formulated (Rascovsky 2011). It is important for non-specialists to have a workable framework for suspecting FTD as it can be particularly challenging to diagnose (Rascovsky 2011).

Neuropsychiatric inventory (NPI) scores are taken during BDR clinical assessments; however, over half the BDR cases were missing these scores, suggesting NPIs are not being utilised as much as they could during assessments and resulting in potentially missed cases of FTD and DLB.

Pathologically, the diagnosis for the control, and AD groups were well matched to the clinical diagnosis. This could imply the assessments are best suited in distinguishing controls and AD. A previous study by Beach et al. (2012) looked at the sensitivity and specificity of the clinical diagnosis of AD against the neuropathological diagnosis, they found sensitivity for clinical diagnosis was increased with more permissive clinical criteria and specificity was increased with more restrictive clinical criteria. The study carried out by the National institute of aging (NIA), for the Alzheimer’s Disease Cooperative Study (ADCs), found they were more accurate when they diagnosed AD, among dementia subjects, than when they diagnosed subjects with other dementing diseases which included; AGD, FTLD, CVD and DLB.

These studies have found uncertainties in diagnosing mixed dementia during life which remain a major limitation in advancing research on the relationship between AD and CVD, limiting the precision of diagnostic decisions.

It is also important to consider that the clinical diagnoses provided by memory services/hospital teams or GPs are not readily available to BDR assessment teams unless disclosed by the next of kin or participant. The BDR assessments occur over an average duration of 8 years; however, missed assessments, and missed questions can be expected due to the nature of the dementing conditions. Additionally, control participants are assessed every 2 years, if they develop a cognitive impairment in those 2 years this may not be picked up by the team.

Future proposals for early detection and diagnosis of dementia patients would increase the likelihood of more accurate diagnosis of dementia subtypes based on observable presentation of DLB, FTD and CVD. The use of more efficient and continued assessments throughout the disease progression would also allow for better care and understanding of the patient based on the dementia subtype.
